# Methicillin-Susceptible *Staphylococcus aureus* in Skin and Soft Tissue Infections, Northern Italy

**DOI:** 10.3201/eid1502.080010

**Published:** 2009-02

**Authors:** Marco Tinelli, Monica Monaco, Maurizio Vimercati, Antonio Ceraminiello, Annalisa Pantosti

**Affiliations:** Hospital of Lodi, Lodi, Italy (M. Tinelli, M. Vimercati, A. Ceraminiello); Istituto Superiore di Sanità, Rome, Italy (M. Monaco, A. Pantosti)

**Keywords:** Staphylococcus aureus, MSSA, Panton-Valentine leukocidin, skin infections, neonatal infections, Italy, research

## Abstract

During February 2004–September 2006, familial clusters and sporadic cases of *Staphylococcus aureus* skin and soft tissue infections were observed in a suburban area near Milan in northern Italy. Molecular typing of the isolates showed an epidemic methicillin-susceptible *S. aureus* (MSSA) strain, *spa* type 005 and sequence type 22 that harbored Panton-Valentine leukocidin (PVL) genes. The first case-patients were neonates or mothers who had recently delivered in the local hospital. Examination of the medical records showed a cluster of postpartum mastitis and neonatal skin infections antedating the emergence of infections in the community**.** Nasal swabs of neonates, mothers, and hospital staff were positive for the epidemic MSSA. Hospital circulation of the strain was interrupted by implementation of infection control measures, although infections continued to occur in the community. The PVL-positive MSSA strain resembles typical community-acquired methicillin-resistant *S. aureus* in its ability to cause prolonged community and hospital outbreaks of skin infections.

## Abstract 2

Summary statement: A community outbreak with intrafamilial skin infections was associated with an MSSA clone.

Long established as a hospital pathogen, methicillin-resistant *Staphylococcus aureus* (MRSA) is now present in the community as a major cause of skin and soft tissue infections (*1*). In the United States the community-acquired (CA)–MRSA clone designated USA300 has been identified in almost 50% of community-onset skin infections (*2*). In Europe, CA-MRSA infections appear to be less common than in the United States, although incidence is increasing (*3**,**4*) and CA-MRSA strains are more genetically diverse (*5*).

Characteristically, most CA-MRSA strains contain Panton-Valentine leukocidin (PVL) genes, a bicomponent pore-forming toxin with the ability to lyse leukocytes (*6*), primarily associated with skin infections such as furunculosis and skin abscesses (*7*). Although clinicians are currently concerned primarily with CA-MRSA infections, methicillin-susceptible *S. aureus* (MSSA) infections can present with similar epidemiologic and clinical characteristics (*8**,**9*). In addition, the presence of PVL genes is not limited to MRSA nor is their presence a recent occurrence. Historical MSSA isolates, such as the “Oxford Staphylococcus” and phage type 80/81 strains that were pandemic in the 1950s and 1960s, harbor PVL genes (*10**,**11*). Recently, PVL-positive MSSA strains have been associated with outbreaks of skin infections in Swiss schoolchildren (*12*), in a village in Germany (*13*), and in French soldiers operating in Côte d’Ivoire (*14*). According to a large multinational clinical trial, conducted outside the United States, PVL-positive *S. aureus* isolates are more likely to be MSSA than MRSA (*15*). The epidemiology of PVL-positive MSSA is not well known and the pathogenic potential is probably underestimated.

This report describes a large and prolonged community and hospital outbreak of skin and soft tissue infections caused by a PVL-positive MSSA strain. In a number of characteristics, the outbreak closely resembles outbreaks associated with CA-MRSA.

## Methods

### Setting of the Community Outbreak

During February 2004–September 2006, several family clusters of skin and soft tissue infections were observed in a suburban area south of Milan in northern Italy. The patients lived in or near the town of Codogno and sought treatment at the outpatient clinic of the local hospital. In each of the family clusters, the first identified case was a newborn child or a mother who had recently delivered in the Codogno Hospital. In 2006, another cluster of skin and soft tissue infections was observed in young patients who lived in different households in the same suburban area.

### Setting of the Hospital Outbreak

Medical records and microbiologic data regarding skin and soft tissue infections occurring in neonates or mothers examined in the Codogno Hospital from 2003 through 2005 were reviewed. At the time of the review, the hospital was a 220-bed facility, serving a community of ≈15,000 inhabitants. The maternity ward comprised 7 rooms with a total of 13 beds and was part of the Department of Obstetrics and Gynecology. The department had 45 staff members that included obstetricians, midwives, nurses, and support staff. Annually, ≈600 deliveries were performed. The newborn nursery was under the direction of the Department of Pediatrics and included 2 rooms, 1 room for changing and feeding the babies and the other room with 14 cribs for the neonates. The nursery staff included 9 neonatal nurses and 1 general nurse, but 16 additional staff members were shared across the Department of Pediatrics. The median length of stay of neonates in the nursery was 4 days. A follow-up visit was performed at a dedicated hospital clinic 10–15 days after discharge.

### Microbiologic and Molecular Typing Methods

Bacteriologic specimens were obtained for culture from the largest infected body area with a sterile swab or needle and were processed in the microbiology laboratory of the Codogno Hospital according to standard methods. Swabs obtained from anterior nares were plated directly onto salt mannitol agar plates and blood agar plates without a preenrichment step. Plates were incubated for 24 h in ambient air and 5% CO_2_, respectively. Identification of isolates and antimicrobial susceptibility tests were performed with an automatic system (Vitek 2; bioMérieux, Marcy l’Etoile, France). The susceptibility pattern was confirmed by the disk-diffusion method following Clinical and Laboratory Standards Institute guidelines (*16*).

Further molecular tests and genotyping were performed on all *S. aureus* isolates that had been stored. Bacterial DNA was prepared with a commercial kit (QIAamp DNA Mini Kit; QIAGEN GmbH, Hilden, Germany). Species identification and methicillin susceptibility were confirmed by a duplex PCR assay with primers targeting *nuc* and *mecA* genes, respectively. Detection of the presence of the genes *lukS-PV* and *lukF-PV* coding for the 2 subunits of the PVL toxin was obtained by PCR (*17*).

To analyze clonal relatedness of the strains, all available isolates were submitted to pulsed-field gel electrophoresis (PFGE) and to a sequence-based method that detects variations in the short sequence repeat (SSR) region of the protein A gene (*spa* typing) (*18*). For PFGE, total genomic DNA embedded in agarose plugs was digested with *Sma*I and separated by using a previously described method (*19*). Profiles of strains that differed by fewer than 3 bands were considered to belong to the same PFGE type (*20*). For *spa* typing, PCR amplification of the SSR region was performed according to the protocol of Shopsin et al. (*18*). Sequences were obtained and analyzed by using an Internet-based software Ridom Staph Type (www.ridom.de/spaserver). Selected isolates underwent multilocus sequence typing (MLST) according to the recommended method (*21*). The allelic profiles obtained were compared with those deposited in the MLST database (http://saureus.mlst.net).

### Screening for *S. aureus* Carriage

To ascertain the spread of *S. aureus* in the maternity ward and nursery of the hospital, we performed screening for *S. aureus* nasal colonization on neonates, mothers, and medical and nonmedical staff who worked in the Departments of Pediatrics and Obstetrics and Gynecology. Two separate surveys were performed, the first in July 2005 and the second in December 2005, after implementation of enhanced infection control measures.

## Results

### Skin and Soft Tissue Infections in the Community

Five familial clusters of skin and soft tissue infections, involving 2–5 family members, were observed in the Codogno area. The first case in each cluster occurred in 2004 or early 2005, but infections in other household members or recurrent infections continued to be observed until 2006. In all families, the onset of infection was associated with a neonate born in the Codogno Hospital or a mother who had recently delivered in the same hospital.

Furunculosis and abscesses were the most common clinical features and relapses were common (Table 1). Furunculosis of the prepuce developed in a neonate in family 1 four days after birth in the Codogno Hospital in February 2004. His father sought treatment for recurrent subcutaneous axillary abscesses later in 2004 and in 2005, and a leg abscess developed in his mother in September 2006.

**Table 1:** Characteristics of patients with community-acquired MSSA skin and soft tissue infections, their treatment, and molecular typing of the isolates, northern Italy, 2004–2006*

Patient	Age, sex	Site of infection†	Type of infection†	Antimicrobial drug treatment†	Drainage	Molecular typing of MSSA isolates			
						Presence of PVL genes	PFGE type	*spa* type	ST
Family clusters									
Cluster 1									
P 1	32 y, F	Leg	Abscess	None	Spontaneous	NA			
P 2	33 y, M	Axilla	Abscesses	AMC, CIP	None	NA			
P 3	4 d, M	Prepuce	Pustules	GEN	None	NA			
Cluster 2									
P 4	30 y, F	Vulva, thighs	Pustules, abscesses	AMC, LFX, TEC	None	+	A	t005	22
P 5	33 y, M	Nose, scalp	Pustules	AMC	None	NA			
P 6	14 mo, F	Thigh	Pustules	CLI	None	NA			
P 7	14 d, F	Thigh	Pustules	CLI	None	+	A	t005	ND
Cluster 3									
P 8	32 y, F	Face, vulva, leg	Abscesses	AMC, CIP, LFX	None	+	A	t005	22
P 9	25 mo, F	Leg	Abscess	None	Spontaneous	NA			
Cluster 4									
P 10	34 y, F	Axilla, forearm, leg	Abscesses	AMC	None	NA			
P 11	35 y, M	Axilla, forearm, leg	Abscess, furuncles	AMC, LFX	Surgical	+	A	t005	ND
P 12	4 d, M	Neck, groin, axilla,	Pustules, abscesses	AMC, AMC	Spontaneous	+	A	t005	22
P 13	3 y, M	Forearm	Abscesses	AMC	None	NA			
P 14	65 y, F	Axilla, forearm, leg, face	Abscess	None	None	NA			
Cluster 5									
P 15	33 y, F	Face, leg, axilla	Pustules, abscess	AMC	Surgical	NA			
P 16	36 y, M	Thigh	Furuncles, abscess	AMC	None	NA			
Sporadic cases									
P 17	64 y, F	Axilla	Abscess	CIP	None	+	A	t005	22
P 18	7 mo, F	Arm	Pustules	AMC	None	–	F	t159	ND
P 19	9 y, F	Axilla	Furuncles	AMC	None	–	F	t159	ND
P 20	12 mo, M	Groin	Abscess	AMC	None	+	A	t005	ND
P 21	8 y, F	Leg	Abscess	AMC	None	–	G	t445	ND
P 22	18 mo, M	Forearm	Furuncles	AMC	None	+	A	t005	ND
P 23	12 mo, F	Buttock	Abscess	AMC	Spontaneous	+	A	t005	ND
P 24	20 mo, F	Thigh	Abscess	AMC	None	+	A	t005	22
P 25	8 y, M	Arm, chest	Abscesses	AMC	Surgical	+	A	t005	ND
P 26	11 y, M	Face, eye	Abscess, conjunctivitis	AMC	None	+	A	t005	22

*MSSA, methicillin-susceptible *Staphylococcus aureus*; PVL, Panton-Valentine leukocidin; PFGE, pulsed-field gel electrophoresis; ST, sequence type; NA, isolate not available; AMC, amoxicillin-clavulanic acid; CIP, ciprofloxacin; GEN, gentamicin (topical); LFX, levofloxacin; TEC, teicoplanin; CLI, clindamycin (topical); ND, not determined. †When >1 site or type of infection or antibimicrobial agents are indicated, they refer to different infection episodes.

The mother in family 2 sought treatment for an infection in the vulva, groin, and inner thighs in August 2004, ten days after delivering at the Codogno Hospital. Several recurrences of abscesses in the same areas occurred as well as in this patient’s left buttock and leg until 2006. In 2005, furunculosis developed in the father on his nose and scalp, and recurring pustules developed on the inner thighs of 2 siblings over 2 months.

In family 3, a subcutaneous facial abscess developed in the mother in January 2005, two months after she delivered at the Codogno Hospital; subsequently, a vulvar abscess and recurrent abscesses of the right leg continued to develop in this patient until 2006. The child had a leg abscess in June 2006.

Family cluster 4 involved 5 persons: a neonate, a sibling, both parents, and the maternal grandmother. Pustules developed on the neck and groin of the neonate 4 days after birth at the Codogno Hospital in January 2005 and subsequently, subcutaneous abscesses developed in the axilla and forearm. Over the same time multiple subcutaneous abscesses developed in the axillae and on the forearms and legs of both parents and the elder sibling. The grandmother had a facial abscess in July 2005, and the father contracted furunculosis of the left forearm in September 2006.

Family cluster 5 involved both parents of a child who was born in the Codogno Hospital in December 2004 but who did not experience skin infections. Facial pustules developed in the mother in February 2005, two months postpartum. Subsequently, she was treated for a leg abscess and axillary furunculosis. In March 2006, a subcutaneous abscess developed in the thigh of her husband in March 2006.

All cases with a bacteriologic diagnosis were caused by MSSA that showed a distinct susceptibility pattern: resistant to penicillin and gentamicin and susceptible to oxacillin, erythromycin, tetracycline, rifampicin, and ciprofloxacin. Nasal swabs were performed in 7 patients from the clusters and were positive for MSSA in 6.

During March–June 2006, eleven additional cases of *S. aureus* skin and soft tissue infections occurred in patients who lived in the same area in different households and who were not related to the family clusters. With the exception of a case caused by CA-MRSA/ST8 in an infant 3 years of age (*22*), the other 10 cases were caused by MSSA. Nine patients were children ranging in age from 6 months to 11 years. Two infants (Table 1, patients 20 and 23) had experienced a pustular rash 1 year earlier, soon after birth in the Codogno Hospital. Clinical signs included pustulosis or furunculosis (3 patients) and abscesses (7 patients). One patient also had conjunctivitis. Patients were neither immunosuppressed nor had preexisting skin infections or other risk factors.

Antimicrobial drugs, mostly oral amoxicillin-clavulanic acid, were given to patients based on their clinical conditions and site and size of the infected area (*23*) (Table 1). A recurrence of a large subcutaneous abscess in 1 adult patient led to treatment with intravenous teicoplanin. Spontaneous drainage occurred in 4 abscesses, and surgical incision and drainage were performed in 3 cases. All patients had a favorable outcome.

### Molecular Typing of Isolates from Community Infections

Isolates available for molecular typing included 5 MSSA from family clusters 2, 3, and 4, and 10 MSSA from the sporadic cases in 2006. All isolates from the family clusters and 7 of the 10 isolates from the sporadic cases contained PVL genes. By PFGE, all PVL-positive isolates appeared indistinguishable or closely related (differing by 1–2 bands) and were assigned to PFGE type A. These isolates also exhibited an identical *spa* type, corresponding to t005. MLST of 6 representative isolates yielded sequence type (ST) 22 (Table 1). Isolates with these characteristics will be subsequently referred to as the “epidemic MSSA clone.” Three PVL-negative MSSA isolates, obtained from infections in 2006, showed different PFGE, *spa*, and MLST types (Table 1).

### Skin and Soft Tissue Infections in Mothers and Neonates

Examination of medical records and microbiologic data from the Codogno Hospital showed a cluster of postpartum mastitis involving 13 women that had occurred from October 2003 through January 2004, before the family clusters were identified. The women had delivered in the same hospital 2–12 weeks before the onset of symptoms. In 6 case-patients, mastitis had progressed to breast abscesses and required surgical drainage. Culture of the drainage yielded MRSA in 1 case-patient and MSSA in the other case-patients. MSSA isolates had a susceptibility pattern identical to that of the community-acquired MSSA. Molecular studies were not performed on these isolates.

In early 2004, several cases of skin infections (mainly pustulosis of the groin or upper thigh) were observed in neonates in the hospital nursery or after discharge when they were examined during routine follow-up visits. From January through March 2004, 14 such skin infections were observed. No other cases were identified until December 2004, when 9 cases occurred. From January through September 2005, skin infections developed in a total of 65 neonates, with peak incidence in June and July when 14 and 17 cases were identified, respectively. In July 2005, screening for nasal *S. aureus* carriage was initiated, and infection control measures were enforced (see Infection Control Measures). Cases of infection gradually diminished, and no new cases were observed after September 2005. MSSA isolates were obtained from all neonates whose specimens had been cultured with skin infections in 2004 and 2005, but the isolates were not stored and were not available for molecular studies.

### Screening for *S. aureus* Carriage and Molecular Typing of Carriage MSSA Isolates

To ascertain the circulation of *S. aureus* in the Codogno Hospital, screening for *S. aureus* nasal carriage was performed in the maternity ward and the nursery in July 2005 and in December 2005, after implementation of infection control measures. In July 2005, nasal swabs were obtained from 48 neonates, 58 mothers, and 71 medical and nonmedical personnel. MSSA was isolated from 19 (39.6%) of 48 neonates in the nursery, 16 (27.6%) of 58 mothers, and 19 (26.8%) of 71 staff (Table 2). No MRSA strain was identified. Remarkably, all of the 17 available isolates from neonates were PVL-positive and 16 corresponded to the epidemic MSSA clone. Only 1 maternal and 3 staff isolates corresponded to the epidemic MSSA clone. Personnel colonized with the epidemic MSSA included 1 pediatrician, 1 newborn nurse, and 1 nurse in the Department of Obstetrics and Gynecology. One of the neonates was colonized by a PVL-positive MSSA that had a PFGE profile, *spa* type, and MLST that were completely different from those of the epidemic MSSA. MSSA isolates that were PVL-negative were genotypically heterogeneous, showing a number of different PFGE profiles and *spa* types (data not shown).

**Table 2:** Results of nasal carriage screenings and molecular typing of PVL-positive MSSA isolates, northern Italy, 2005*

Persons sampled (no.)	MSSA carriers, no. (%)	MSSA isolates			PVL-positive isolates		
		No. examined	No. PVL+		PFGE type (no. isolates)	*spa* type	ST
July							
Neonates (48)	19 (39.6)	17	17		A (16)	t005	22
					B (1)	t021	956
Mothers (55)	16 (27.6)	10	1		A (1)	t005	22
Staff (71)	19 (26.8)	17	4		A (3)	t005	22
					A (1)	t2336	954
December							
Neonates (43)	0	0	0				
Mothers (17)	5 (29.4)	3	1		A (1)	t005	22
Staff (64)	3 (4.7)	3	2		D (1)	t645	1210
					E (1)	t1445	1209

*PVL, Panton-Valentine leukocidin; MSSA, methicillin-susceptible *Staphylococcus aureus*; PFGE, pulsed-field gel electrophoresis; ST, sequence type.

In the December 2005, screening, nasal swabs were obtained from 43 neonates, 17 mothers, and 64 staff. No neonate was colonized with *S. aureus*, although 5 (29.4%) of 17 mothers and 4 (6.2%) of 64 staff members carried MSSA. In addition, a staff member was colonized with MRSA. Only 1 isolate from a mother corresponded to the epidemic PVL-positive MSSA clone, which suggests that although transmission inside the nursery had been interrupted, the epidemic strain was still circulating in the community. Two pediatric nurses were colonized by PVL-positive MSSA isolates that showed PFGE types, *spa* types, and STs that were unrelated to the outbreak MSSA clone (Table 2).

In all cases but 1, results of *spa* typing were in accordance with the PFGE analysis, by clustering the isolates belonging to the epidemic clone and discriminating genetically different isolates. The exception was a PVL-positive MSSA isolate obtained from a neonatal nurse in the July screening. This isolate was PFGE type A that had a novel *spa* type (t2336) resulting from deletion of 4 of the 12 repeats of t005, and yielded a novel combination of MLST alleles (ST954) that was a single locus variant of ST 22.

### Infection Control Measures

When the neonatal outbreak of skin infections was identified in December 2004, contact precautions were instituted in the nursery and the maternity ward for staff and mothers, who were required to wear a gown and mask when feeding their babies. In June 2005, these control measures were expanded to include enhanced contact precautions based on existing recommendations to control the spread of MRSA and other drug-resistant microorganisms (*24*). Notices to promote handwashing among personnel as well as among mothers and visitors were posted on the walls of the nursery and waiting areas. The nursery and adjacent areas were deeply cleaned with chlorine-containing disinfectant. After nasal carriage screening in July 2005, intranasal mupirocin was administered to all neonates and was continued until hospital discharge, usually for 5 days. Personnel and mothers with MSSA-positive nasal cultures received nasal mupirocin for 5 days with or without a 3-day course of amoxicillin/clavulanate. After the introduction of these measures, cases of MSSA skin infections in neonates decreased and no new cases occurred in the hospital after September 2005, no infections were reported at follow-up visits, and no neonates in the nursery carried *S. aureus* in the December 2005 nasal screening.

## Discussion

We have described a large and prolonged outbreak caused by a PVL-positive MSSA strain that was probably initiated in the maternity ward and nursery of the local hospital and spread to the community. Striking similarities exist between the principal features of this outbreak and recent descriptions of outbreaks caused by typical CA-MRSA. First, skin infections occurred predominantly in children and young adults without risk factors, with intrafamilial spread and recurrences (several examples of familial transmission of CA-MRSA have been described in which family members can serve as a reservoir of CA-MRSA) (*25**,**26*). Second, the epidemic MSSA clone was prolonged in the community (in northern Denmark, a CA-MRSA strain was responsible for a community outbreak of recurring infections that involved 46 persons >6 years of age) (*27*). Third, a mastitis outbreak occurred and neonatal infections emerged in the local hospital (an outbreak of CA-MRSA infections in a neonatal intensive care unit was likely initiated by the mother of the index case who had a CA-MRSA wound infection and mastitis) (*28*). Twenty-one percent of neonates with CA-MRSA infections at the Texas Children’s Hospital had a mother with a history of skin infections, including mastitis and axillary abscesses (*29*).

Our study has some clear limitations because the PVL-positive MSSA outbreak strain was only demonstrated in isolates from 3 family clusters, in community infections in 2006, and in the hospital carriers. That the epidemic clone was responsible for the other 2 family clusters and the postpartum mastitis outbreak can only be inferred from the records of isolation of MSSA with a distinctive susceptibility pattern (i.e., resistant to penicillin and gentamicin only). As for infections in neonates, these were generally considered mild, and microbiologic cultures were performed in only a few cases. In addition to the microbiologic findings, epidemiologic and clinical data support the presence of an unusually virulent strain. In the Codogno Hospital outbreak, the temporal relationship between the mastitis outbreak and the emergence of neonatal MSSA infections suggests that the source of the strain might have been a mother who had undetected infection or colonization at delivery and transmitted the strain to the baby. Subsequently, the strain spread inside the newborn nursery, possibly with the contribution of colonized healthcare workers, leading to the colonization of babies and the emergence of skin infections a few days after birth. The colonized/infected mothers and neonates in turn spread the MSSA strain in the community.

Implementation of infection control measures, including enhanced hand hygiene, contact precautions, and mupirocin treatment, resulted in a rapid decline in the occurrence of neonatal infections and the disappearance of the strain among neonatal carriers. There are no established data to support prophylactic treatment with mupirocin in MSSA-colonized patients, although its use has been proposed for some colonized at-risk patients who will undergo surgery (*30*). Despite control in the hospital, skin infections caused by the epidemic MSSA clone continued in family clusters, and new cases unrelated to family clusters were recognized in 2006. In the family clusters, hygienic measures were suggested to avoid spread to other family members, but decolonization with mupirocin was not attempted. Two recent reports highlight the efficacy of mupirocin decolonization to terminate outbreaks of skin infections in the community (*12**,**13*).

Since June 2006, only 2 new cases caused by the epidemic MSSA strain were observed in 2 adult men in the community, 1 in 2007 and the other in 2008, indicating that the outbreak was controlled but that the strain had not disappeared from the community. In addition, in July 2007, the father in family cluster 1 experienced a recurrence of a chest abscess caused by the epidemic MSSA strain. Although no earlier isolate from that family was studied, the same epidemic strain was likely responsible for skin infections in that family over the span of at least 3 years.

The PVL-positive outbreak MSSA strain, characterized by t005 and ST22, is related to one of the major MRSA clones circulating in hospitals in the United Kingdom, where it has been designated EMRSA-15 (*31*). Although this clone, also known as ST22-IV, harbors the type IV staphylococcal cassette chromosome *mec* (SCC*mec*) and is uncommon in the United States (*32*), it is now emerging as a successful clone in several areas of the world (*33**,**34*). Characteristically EMRSA-15 is susceptible to gentamicin and resistant to erythromycin and ciprofloxacin (*34*).

Clonal group ST22 includes MRSA as well as MSSA (*35*), both of which can contain PVL genes. Recently, a PVL-positive ST22 MRSA strain caused a large outbreak in Bavaria (*36*). This strain was susceptible to most non–β-lactam antimicrobial agents, including erythromycin and ciprofloxacin. The MSSA strain responsible for the outbreak in northern Italy was susceptible to erythromycin and ciprofloxacin and resistant to gentamicin, resulting in a susceptibility profile highly divergent from that of EMRSA-15. The epidemic MSSA clone could represent the ancestor of a hospital MRSA clone or, less likely, a derivative of a hospital MRSA clone that emerged by deletion of SCC*mec*. Whichever is the case, PVL genes have been introduced into a genetic background associated with the ability to spread rapidly and cause epidemics.

The role of PVL in the pathogenesis of *S. aureus* infections is still controversial. Animal models of necrotizing pneumonia provide conflicting results (*37**,**38*). In a mouse model of skin infections, PVL did not seem to play an indispensable role (*39*). However, it is difficult to dismiss the simple observation that *S. aureus* isolates causing skin infections in humans are enriched for PVL (*40*). This is more striking in Europe, where PVL is associated with a variety of different *S. aureus* genotypes (*5*) and not with a single major clone as in the United States (*2*). Hypothetically, PVL could play a role that has not been explored in animal models, e.g., to enhance persistence in nasal colonization or survival on the skin. Alternatively, the PVL bacteriophage could confer other properties to *S. aureus* that contribute to the pathogenesis of skin infections.

The presence of PVL or the PVL bacteriophage may contribute to some of the characteristics of this clone that are shared with typical CA-MRSA, including its ability to persist in the human reservoir, to cause skin infections in healthy young persons, and to require enhanced infection control precautions in the hospital. The only distinctive difference with CA-MRSA infections is the wider spectrum of therapeutic options available that includes β-lactam antimicrobial agents. On the other hand, the hospital and community outbreaks were initially overlooked because the causative agent of the infections was an MSSA strain. This study underscores how the overall genetic background of the *S. aureus* strain and not the methicillin resistance trait per se, determines clinical severity and the epidemiologic features of infections.
